# Challenges in Management of Rhegmatogenous Retinal Detachment in a Patient With Wilson’s Disease: A Case Report and Literature Review

**DOI:** 10.7759/cureus.12921

**Published:** 2021-01-26

**Authors:** Waleed M Alkhuraimi, Wijdan Alqahtani, Abdullah S Alqahtani

**Affiliations:** 1 Ophthalmology, King Abdullah Medical City, Mecca, SAU; 2 Ophthalmology, King Khalid University, Abha, SAU; 3 Ophthalmology, King Abdulaziz Medical City, Ministry of National Guard Health Affairs, Jeddah, SAU; 4 Ophthalmology, King Saud Bin Abdulaziz University for Health Sciences, Jeddah, SAU

**Keywords:** wilson’s disease, rhegmatogenous retinal detachment, proliferative vitreoretinopathy, pan-retinal photocoagulation

## Abstract

Wilson’s disease (WD) is a rare hepatolenticular inherited disorder affecting copper transport resulting in accumulation of copper, which leads to the induction of apoptosis in different organs. Furthermore, patients with WD have elevated cytokines activity responsible for inflammation of various tissues.

Here, we report our challenges in managing a case of rhegmatogenous retinal detachment (RRD) in a one-eyed 28-year-old male with WD who had a previous history of severe intraocular inflammation that ended with phthisis bulbi after pars plana vitrectomy for RRD. After one year, he developed RRD in the seeing eye.

A decision was made to perform scleral buckling to avoid the risk of postoperative intraocular inflammation. However, a barrage laser was required for shallow retinal detachment in a subsequent follow-up, which was ultimately complicated by severe intraocular inflammation.

We observed that our patient with WD had a tendency for severe intraocular inflammation, even following minor non-surgical ophthalmic procedures. For this reason, ophthalmologists need to be aware of managing similar cases and perhaps other diseases associated with elevated levels of cytokines.

## Introduction

Wilson’s disease (WD) is a rare autosomal recessive genetic disorder that affects copper metabolism and leads to copper accumulation in the liver, brain, eye, and other organs. *ATP7B *is the causative gene, with more than 500 identified mutations [1]. WD has established ocular manifestations, including Kayser-Fleischer corneal rings, sunflower cataract, and, rarely, night blindness, optic neuritis, optic disc pallor, and exotropia [2].

Patients with WD have increased activity of inflammatory cytokines with resultant manifestations such as hepatitis and neuroinflammation [3]. Furthermore, increased free radical formation and oxidative stress secondary to high intracellular concentrations of copper lead to induction of apoptosis of hepatocellular, neuronal, and other tissues [1].

For any patient with rhegmatogenous retinal detachment (RRD), the most dreaded sequelae is proliferative vitreoretinopathy (PVR), which results in scarring and membrane formation of the retina and vitreous secondary to inflammatory cytokines. PVR carries a significant risk for failure of retinal detachment surgery [4] and has well-known risk factors including an untreated RRD, post-pneumatic retinopexy, laser retinopexy, scleral buckle (SB), or pars plana vitrectomy (PPV) [5].

We report the unusual manifestations and challenges faced managing an RRD in a patient with WD who had a history of severe intraocular inflammation and phthisis bulbi following PPV of his non-presenting eye.

## Case presentation

**Figure 1:**
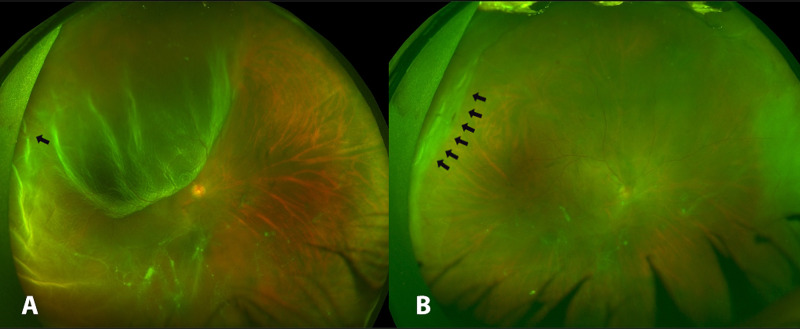
Wide-field fundus photos of the right eye showing (A) corrugated retinal detachment involving the macula with flap tear (black arrow). (B) Flat retina post-SB; note SB indentation (black arrows). SB, scleral buckling

A one-eyed 28-year-old male with WD who was in a state of immunologic tolerance following a liver transplantation presented to the emergency department of our institute complaining of sudden, painless decreased vision of his only seeing right eye associated with flashes and a curtain-like shadow. His vision in the right eye despite correction was counting fingers (CF), and dilated fundus examination (DFE) showed a “macula off” superotemporal RRD extending to the inferotemporal quadrant with a nearby horseshoe retinal tear (Figure 1A).

Ophthalmic history revealed our patient had been following regularly at our clinic for moderate-to-high myopia (spherical equivalent of -6.50 Diopters and -5.00 Diopters in the right presenting and left non-seeing eyes, respectively) and had a previous RRD in the left non-seeing eye one year prior to his current presentation, which was managed with PPV and was complicated by severe postoperative inflammation and ultimately ended with phthisis bulbi despite appropriate and timely care. Family history showed that our patient was born to consanguineous parents with five siblings, all of whom were myopes, with a similar occurrence of an RRD in one of his brothers who refused any intervention.

**Figure 2:**
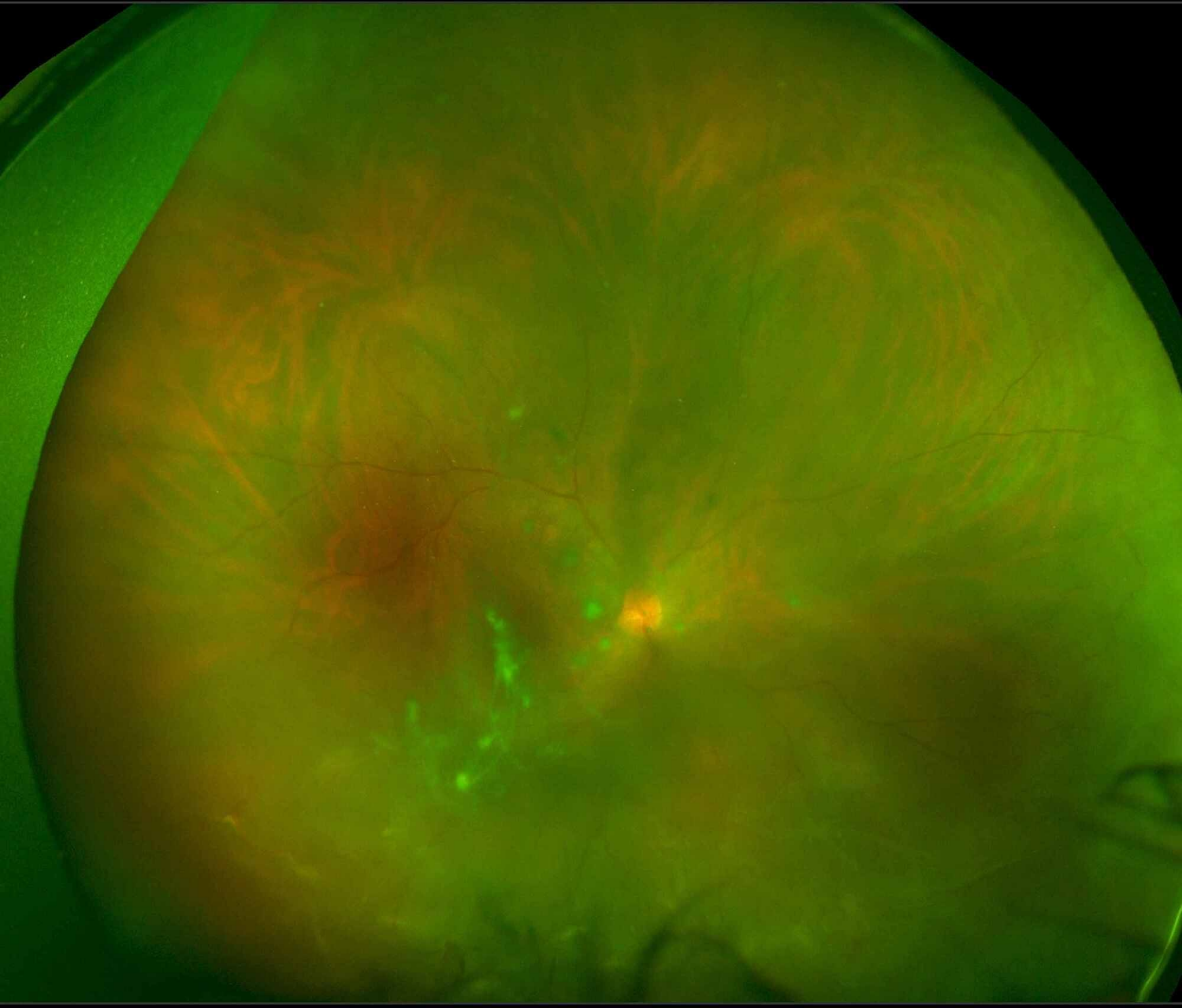
Wide-field fundus photo of the right eye showing grade A PVR. PVR, proliferative vitreoretinopathy

It was decided that intraocular surgery should be avoided to minimize the risk of postoperative inflammation as transpired in the other eye. For this reason, he underwent urgent SB that was uneventful for the repair of his RRD, and he was discharged on antibiotics and a tapered dose of topical steroids four times a day for one month. Three weeks postoperatively, the vision with correction had improved to 20/80 and DFE showed a flat retina (Figure 1B). Two months following the initial surgery, a barrage laser with three rows was performed for the appearance of a shallow retinal detachment. Intriguingly, three weeks after barrage laser therapy, his vision started gradually worsening. Best Corrected Visual Acuity was found to be 20/200 and his intraocular pressure (IOP) remained normal. Examination showed diffuse corneal keratic precipitates (KPs) with anterior chamber reaction of 3+ cells and 1+ flare, posterior synechiae, and white cataract with pigments on the anterior lens capsule. The fundus examination showed grade A PVR (Figure 2). Workup for infectious and non-infectious uveitis was performed and all investigations were unremarkable. The patient was started on a topical steroid to control inflammation.

Multiple appointments were missed by the patient, but he eventually returned to the clinic complaining of decreased vision, halos, and pain in the right eye. Vision with correction was found to be CF and his IOP was 60 mmHg. Anterior segment examination revealed extensive posterior synechia, iris bombe, and a white cataract. Consequently, a diagnosis of secondary angle closure was made and the patient underwent a laser peripheral iridotomy, which was successful in reducing his IOP to 12 mmHg with the aid of aqueous suppressants as well as the topical steroid for intraocular inflammation control.

## Discussion

Inflammatory cytokines tend to be elevated in WD. A recent study of 99 patients with WD showed elevated levels of inflammatory cytokines compared to healthy individuals [3]. In addition, patients with WD have a risk of copper depletion as a side effect of copper-chelating agents, which ultimately affect the normal cellular function of mitochondrial biogenesis in retinal pigment epithelium (RPE) cells [6].

PVR is the most common cause of failed RRD surgeries, with an incidence ranging from 5.1% to 11.7% [7]. It is a cellular proliferation creating a contractile membrane composed of RPE cells, glial cells, fibroblasts, and inflammatory cells [5]. Among these cellular components, RPE cells are predominantly involved in PVR membrane formation through an epithelial mesenchymal transition, which is a process of RPE cells transforming from static polarized epithelial cells to proliferative, migratory spindle cells after exposure to inflammatory cytokines in RRD [4,5]. Histologically, PVR is classified into three types: epiretinal, subretinal, and intraretinal PVR [4].

Studies found no association between myopia (including high myopia) and postoperative PVR [5]. However, it may occur in untreated RRD, following pneumatic retinopexy, laser retinopexy, SB, or PPV [5,8]. Nevertheless, a multicentric, observational, case-control study of 335 patients concluded that a significant reduction in the risk of developing PVR might be achieved by PPV, particularly in patients with aphakia and pseudophakia [9]. Furthermore, several pharmacological agents have been used to reduce the risk of developing postoperative PVR, including systemic corticosteroid and intravitreal corticosteroid, low-molecular-weight heparin, and 5-fluorouracil [10-12]. One reported case showed a favorable outcome with the use of a dexamethasone implant with episcleral surgery to reduce the formation of postoperative PVR in case of RRD with a high risk of PVR [13].

There are two main blood-ocular barriers to maintain a desirable intraocular environment by restricting the entry of blood into the eye, blood-aqueous barrier (BAB), and blood-retinal barrier (BRB). The aqueous humor homeostasis is regulated by the BAB which acts as a filter between the vascular bed and aqueous humor. It is formed by the nonfenestrated iris capillaries and the tight junctions of nonpigmented epithelia of the iris and ciliary body [14]. Similarly, the BRB in the RPE’s outer wall and the tight junctions of endothelial membrane of the retinal vessels is responsible for neuroretinal homeostasis [8]. Ocular procedures or disturbed anatomy as in RRD will lead to breakdown of these barriers which trigger undesired intraocular inflammation by exposing the intraocular tissues to specific serum components [8].

A study measuring BAB breakdown following pan-retinal photocoagulation (PRP) in 25 eyes of 25 patients with proliferative diabetic retinopathy (PDR) using a laser flare photometer. The results of this study showed an increase in aqueous flare value after PRP at three, 24, and 48 hours compared to baseline but not at 72, 96, and 168 hours nor eight weeks, with a peak value at 24 hours. However, there was no evidence of clinically significant uveitis on examination [15].

SB results in decreased retinal blood flow, and due to its mechanical pressure on the vortex veins, uveal blood flow will be diminished which may result in disruption of BAB [8]. One reported case of a diabetic patient who underwent PPV and SB was complicated by hypopyon uveitis following PRP for PDR. The author hypothesized that the anterior segment ischemia secondary to SB predisposed patients with diabetes to hypopyon uveitis following PRP. Furthermore, two reported cases in the literature of diabetic patients developed hypopyon uveitis following PRP for PDR. The first patient had uncontrolled diabetes while the second had well-controlled diabetes but had a history of previous uveitis [16,17].

In our case, avoidance of intraocular interventions in the seeing eye was actively sought due to the severe inflammation that ultimately culminated in phthisis bulbi following an uneventful PPV in the nonseeing eye. Therefore, it was decided to perform SB to minimize the inflammatory response postoperatively. However, he developed severe intraocular inflammation following a minor ophthalmic intervention by retinal barrage laser.

We observed that major and even minor ophthalmic procedures may cause a severe ocular inflammatory response in our patient. We probably attribute our patient’s exaggerated inflammatory response and complications to the elevated cytokines in addition to the inherent risks associated with the procedures. Therefore, management of RRD in patients with WD may be challenging and require a careful preoperative, intraoperative, and postoperative evaluation and intervention to prevent and control inflammation with the use of steroids perioperatively and intraoperatively. If required, it may also prove prudent to avoid numerous burns and high power settings when performing retinal laser therapy with close monitoring postoperatively.

## Conclusions

The ophthalmologist needs to be aware that systemic diseases that have increased activity of inflammatory cytokines such as WD may predispose to severe intraocular inflammation following major or minor ocular procedures. Therefore, special consideration is required to prevent severe postoperative inflammation. Further studies are necessary to evaluate the relationship between WD and increased ocular inflammation following ophthalmic procedures. The efficacy of using frequent perioperative steroids and intraoperative steroids in such patients as well as developing a management regimen may prove beneficial for standardization of care and avoidance of complications.

## References

[1] Bandmann O, Weiss KH, Kaler SG (2015). Wilson’s disease and other neurological copper disorders. Lancet Neurol.

[2] Doğuizi S, Özateş S, Hoşnut FÖ, Şahin GE, Şekeroğlu MA, Yılmazbaş P (2019). Assessment of corneal and lens clarity in children with Wilson disease. J AAPOS.

[3] Wu P, Dong J, Cheng N, Yang R, Han Y, Han Y (2019). Inflammatory cytokines expression in Wilson’s disease. Neurol Sci.

[4] Mudhar HS (2020). A brief review of the histopathology of proliferative vitreoretinopathy (PVR). Eye.

[5] Lleó Pérez A, Campos Fernández R, López Santoveña F, Sánchez Lorente G, Hernández Martínez FJ, Navarro Palop C (2000). Clinical risk factors for proliferative vitreoretinopathy after retinal detachment surgery [Article in Spanish]. Arch Soc Esp Oftalmol.

[6] Aloysius Dhivya M, Aberami S, Nikhalashree S (2020). Copper mediates mitochondrial biogenesis in retinal pigment epithelial cells. Biochim Biophys Acta Mol Basis Dis.

[7] Kwon OW, Song JH, Roh MI (2015). Retinal detachment and proliferative vitreoretinopathy. Dev Ophthalmol.

[8] Nagasaki H, Shinagawa K, Mochizuki M (1998). Risk factors for proliferative vitreoretinopathy. Prog Retin Eye Res.

[9] Rodríguez De La Rúa E, Pastor JC, Aragón J (2005). Interaction between surgical procedure for repairing retinal detachment and clinical risk factors for proliferative vitreoretinopathy. Curr Eye Res.

[10] Koerner F, Merz A, Gloor B, Wagner E (1982). Postoperative retinal fibrosis - a controlled clinical study of systemic steroid therapy. Graefe’s Arch Clin Exp Ophthalmol.

[11] Geoff Williams R, Chang S, Comaratta MR, Simoni G (1996). Does the presence of heparin and dexamethasone in the vitrectomy infusate reduce reproliferation in proliferative vitreoretinopathy?. Graefe’s Arch Clin Exp Ophthalmol.

[12] Sundaram V, Barsam A, Virgili G (2013). Intravitreal low molecular weight heparin and 5-fluorouracil for the prevention of proliferative vitreoretinopathy following retinal reattachment surgery. Cochrane Database Syst Rev.

[13] Reibaldi M, Russo A, Longo A (2013). Rhegmatogenous retinal detachment with a high risk of proliferative vitreoretinopathy treated with episcleral surgery and an intravitreal dexamethasone 0.7-mg implant. Case Rep Ophthalmol.

[14] Ragg S, Key M, Rankin F, WuDunn D (2019). The effect of molecular weight on passage of proteins through the blood-aqueous barrier. Investig Ophthalmol Vis Sci.

[15] Moriarty AP, Spalton DJ, Shilling JS, Ffytche TJ, Bulsara M (1996). Breakdown of the blood-aqueous barrier after argon laser panretinal photocoagulation for proliferative diabetic retinopathy. Ophthalmology.

[16] Sinha MK, Narayanan R, Chhablani JK (2014). Hypopyon uveitis following panretinal photocoagulation in a diabetic patient. Semin Ophthalmol.

[17] Tyagi M, Ambiya V, Rani PK (2016). Hypopyon uveitis following panretinal photocoagulation. BMJ Case Rep.

